# Circulating mitochondrial dysfunction as an early biomarker for contrast media‐induced acute kidney injury in chronic kidney disease patients

**DOI:** 10.1111/jcmm.17806

**Published:** 2023-06-12

**Authors:** Prit Kusirisin, Nattayaporn Apaijai, Kajohnsak Noppakun, Srun Kuanprasert, Siriporn C. Chattipakorn, Nipon Chattipakorn

**Affiliations:** ^1^ Division of Nephrology, Department of Internal Medicine, Faculty of Medicine Chiang Mai University Chiang Mai Thailand; ^2^ Cardiac Electrophysiology Research and Training Center, Faculty of Medicine Chiang Mai University Chiang Mai Thailand; ^3^ Center of Excellence in Cardiac Electrophysiology Research Chiang Mai University Chiang Mai Thailand; ^4^ Cardiac Electrophysiology Unit, Department of Physiology, Faculty of Medicine Chiang Mai University Chiang Mai Thailand; ^5^ Division of Cardiology, Department of Internal Medicine, Faculty of Medicine Chiang Mai University Chiang Mai Thailand; ^6^ Department of Oral Biology and Diagnostic Sciences, Faculty of Dentistry Chiang Mai University Chiang Mai Thailand

**Keywords:** cell death, contrast‐induced acute kidney injury, inflammation, mitochondrial dynamics, oxidative stress

## Abstract

Contrast‐induced acute kidney injury (CI‐AKI) is the common hospitalized acute kidney injury (AKI). However, the diagnosis by serum creatinine might not be early enough. Currently, the roles of circulating mitochondria in CI‐AKI are still unclear. Since early detection is crucial for treatment, the association between circulating mitochondrial function and CI‐AKI was tested as a potential biomarker for detection of CI‐AKI. Twenty patients with chronic kidney disease (CKD) undergoing percutaneous coronary intervention (PCI) were enrolled. Blood and urine samples were obtained at the time of PCI, and 6, 24, 48 and 72 h after PCI. Plasma and urine neutrophil gelatinase‐associated lipocalin (NGAL) were measured. Oxidative stress, inflammation, mitochondrial function, mitochondrial dynamics and cell death were determined from peripheral blood mononuclear cells. Forty percent of patients developed AKI. Plasma NGAL levels increased after 24 h after receiving contrast media. Cellular and mitochondrial oxidative stress, mitochondrial dysfunction and decreased mitochondrial fusion occurred at 6 h following contrast media exposure. Subgroup of AKI had higher %necroptosis cells and TNF‐α mRNA expression than subgroup without AKI. Collectively, circulating mitochondrial dysfunction could be an early predictive biomarker for CI‐AKI in CKD patients receiving contrast media. These findings provide novel strategies to prevent CI‐AKI according to its pathophysiology.

## 1 INTRODUCTION

The incidence of contrast‐induced acute kidney injury (CI‐AKI) is relatively low (3%–5%) in patients with normal kidney function undergoing angiography.^1^, ^2^ However, the incidence of CI‐AKI was drastically increased to 40% in patients with impaired kidney function.^1^ Although this is a reversible condition, approximately 15% of the patients need temporary dialysis.^3^ In cases of no renal recovery, it may progress to chronic kidney disease (CKD) or end‐stage renal disease (ESRD) which lead to an increased mortality rate of 3.8%–64%, especially in high risk cases.^3^

Currently, serum creatinine (Cr) is the marker that is well‐accepted for a diagnosis of acute kidney injury (AKI)^4^; however, it is not a sensitive marker of this condition. According to the Kidney Disease Improving Global Outcomes (KDIGO) guidelines, CI‐AKI is diagnosed when one of the following criteria is met (1) an increase in serum Cr > 0.3 mg/dL from their respect baseline within 48 h, or (2) an increase in serum Cr > 1.5‐fold over the baseline within 7 days, (3) a decrease in urine volume < 0.5 mL/kg/h at least 6 h after exposure to contrast media.^4^, ^5^ However, using of serum Cr as a marker of CI‐AKI is often late and with low sensitivity.^1^ Therefore, the early detection of CI‐AKI is important, and is needed to reduce the hospitalization and mortality rate in CKD patients who undergoing angiography.

An increased oxidative stress has been proposed as the principal mechanism of CI‐AKI.^6^ The generation of reactive oxygen species (ROS) suppresses the vasodilation effect of nitric oxide, leading to a sustained vasoconstriction, and contributing to medullary hypoperfusion.^6^ It is known that mitochondria is a main source of ROS production.^7^ Data from animal studies showed that mitochondrial dysfunction plays a crucial role in the development of AKI after contrast media exposure, which can be observed along with increasing oxidative stress and inflammation.^8^, ^9^ Although the information from the direct kidney mitochondrial function is a useful biomarker in CI‐AKI, this procedure is too invasive. Therefore, in the present study we used the peripheral blood mononuclear clear cells (PBMCs) to investigate the effects of contrast media on the oxidative stress, inflammation, mitochondrial function, and cell death, and their associations with the development of CI‐AKI. We hypothesized that mitochondrial dysfunction in PBMCs occurs prior to serum Cr rising, and that it could be used as an early biomarker in CI‐AKI.

## 2 MATERIALS AND METHODS

### 2.1 Study designs

This observational study was conducted at Chiang Mai University Hospital between October 2020 and April 2021. This trial was registered at Thai Clinical Trials Registry (TCTR20210123004), and the protocol was approved by the Research Ethics Committee, Faculty of Medicine, Chiang Mai University (permit no. 422/2020). This research was performed in accordance with the international guidelines for human research protection as Declaration of Helsinki,^10^ The Belmont Report^11^ and International Conference on Harmonization in Good Clinical Practice (ICH‐GCP).^12^ Twenty non‐dialysis CKD patients, which were stage 3–5 with estimated glomerular filtration rate (eGFR) < 60 mL/min/1.73 m^2^ as measured by CKD‐Epidemiology Collaboration (CKD‐EPI) method, undergoing percutaneous coronary intervention (PCI) were included to the study. The exclusion criteria consisted of ESRD on long‐term renal replacement therapy (RRT), AKI with RRT, receiving contrast media within 5 days, acute coronary syndrome within 5 days, acute decompensated heart failure, life‐threatening cardiac arrhythmia (including ventricular tachycardia and ventricular fibrillation), cardiac arrest, cardiogenic shock, sepsis or septic shock, active malignancy, volume of contrast in mL <3 times of eGFR, lupus nephritis, autosomal dominant polycystic kidney disease and severe anaemia with haemoglobin <7 mg/dL. The sample size was calculated from the changes of urine neutrophil gelatinase‐associated lipocalin (NGAL) which was the early biomarker of CI‐AKI in previous study.^13^

The primary outcome of the study was to investigate an effect of contrast media on renal injury, mitochondrial dynamics, mitochondrial function, oxidative stress, inflammation and cell death pathways in CKD patients. The secondary outcomes were investigated all those parameters in the subgroup of AKI and non‐AKI. The definition of AKI was defined according to the KDIGO clinical practice guidelines for AKI: (1) an increase in serum Cr of >0.3 mg/dL over the baseline value within 48 h after exposure to contrast media, (2) an increase in serum Cr >1.5 times over the baseline value within 7 days after exposure to contrast media and (3) a decrease in urinary volume of <0.5 mL/kg/h for at least 6 h after exposure.^4^

### 2.2 Study protocol

**FIGURE 1:**
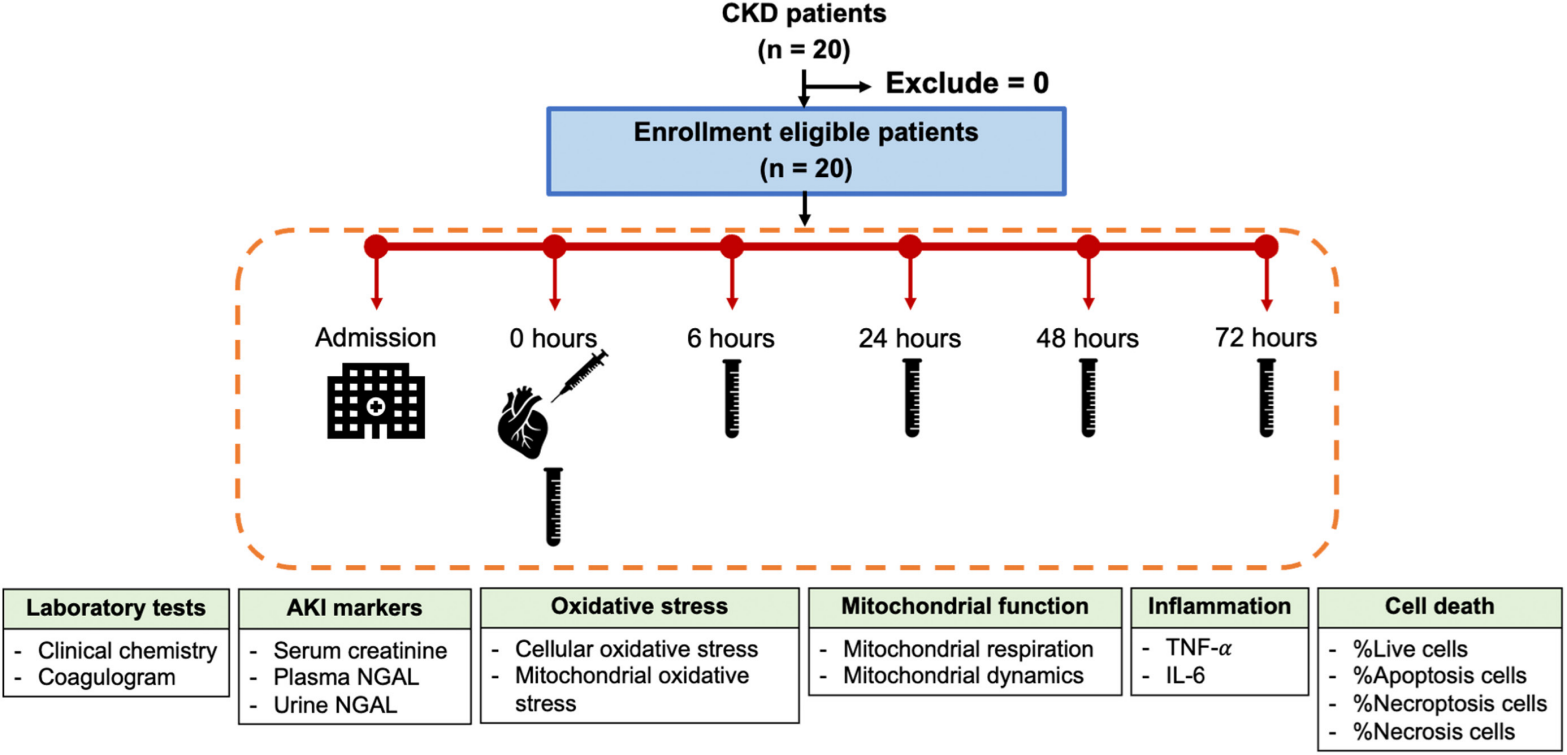
Study diagram of this study. AKI, acute kidney injury; CKD, chronic kidney disease; IL‐6, interleukin‐6; NGAL, neutrophil gelatinase‐associated lipocalin; TNF‐α, tumour necrosis factor alpha.

Eligible patients were assigned to study period for 30 days. All patients obtained verbal and written information concerning the study design, intervention protocol and measurements (blood and urine examination). Patients who agree to the study signed an informed consent. After admission, the blood and urine sample were collected from the patients at five time points including at the time of PCI (baseline), 6, 24, 48 and 72 h after PCI as shown in Figure 1. The type of contrast media was chosen depended on the interventionist.

### 2.3 Renal function parameters and AKI biomarker

Complete blood count, glucose, blood urea nitrogen (BUN), Cr, sodium, potassium, chloride, bicarbonate, calcium, phosphate, magnesium, uric acid, liver function test and coagulogram were determined with routine automated methods at the Central Laboratory of Chiang Mai University Hospital, Faculty of Medicine, Chiang Mai University, Thailand.

Plasma and urine NGAL were measured using a commercial sandwich ELISA kit (human Lipocalin‐2/NGAL ELISA kit, RAB0332, Sigma‐Aldrich).

### 2.4 Peripheral blood mononuclear cell isolation for cellular oxidative stress, mitochondrial oxidative stress, mitochondrial function and cell death analysis

Peripheral blood mononuclear cells (PBMCs) were isolated using a Ficoll density gradient centrifugation as previously described.^14^ PBMCs were used to measure mitochondrial respiration, cellular oxidative stress, mitochondrial oxidative stress and cell death. As brief, the initial centrifugation (1000*g* for 10 min) was performed; white and red blood cells were collected and then re‐suspended in phosphate buffer saline solution (PBS). Subsequently, a diluted white and red blood cells were over‐layered on Ficoll‐Paque reagent (Histopaque, Sigma‐Aldrich) and centrifuged at 400*g* 30 min. After centrifugation, the ring of PBMCs at the Ficoll/plasma interface was collected, and then, the PBMCs were washed with PBS. The viable of PBMCs were stained with trypan blue dye, and cell number was counted by an automatic haemocytometer (NanoEntek).

To determine the levels of cellular oxidative stress levels, 2 × 10^5^ cell of PBMCs was stained with 2 μM of dichloro‐dihydro‐fluorescein diacetate (DCFH‐DA) dye, the fluorescent intensity of DCF was used to indicate cellular oxidative stress levels, which was detected by flow cytometer (BD FACS Celesta).^14^

To determine the levels of mitochondrial oxidative stress, 2 × 10^5^ cell of PBMCs was stained with 5 μM of MitoSOX red dye (Thermo fisher), the fluorescent intensity of MitoSOX was used to indicate mitochondrial oxidative stress levels, which was detected by flow cytometer (BD FACS Celesta).^15^

To determine the levels of cell death, 2 × 10^5^ cell of PBMCs was stained with Annexin V/PI assay kit (BD bioscience). The number of % live cells (Annexin V^−^/PI^−^), %apoptosis cells (Annexin V^+^/PI^−^), %necroptosis cells (Annexin V^−^/PI^+^) and %necrosis cells (Annexin V^+^/PI^+^) was measured by flow cytometry.^16^

To determine mitochondrial respiration, 2 × 10^5^ cell of PBMCs was used to measured oxygen consumption rate (OCR) using extracellular flux analyser (Agilent Seahorse XFe96) with Seahorse XF Cell Mito Stress Test Kit (Agilent Technologies). Briefly, basal respiration was measured in PBMCs with base medium; then, 1 μM of oligomycin was added followed by 2 μM of Carbonyl cyanide‐*p*‐trifluoromethoxyphenylhydrazone (FCCP) and 0.5 μM of rotenone/antimycin A. ATP production, basal respiration, maximal respiration and spare respiratory capacity were reported.^15^

### 2.5 Western blot analysis for mitochondrial dynamics protein expression determination

The protein was extracted from PBMCs using a radioimmunoprecipitation assay (RIPA) buffer. The total protein (0.3 mg/mL) was mixed with loading buffer, loaded onto 10% SDS‐acrylamide gels and then transferred to nitrocellulose membranes in a glycine/methanol‐transfer buffer using a Wet/Tank blotting system (Bio‐Rad Laboratories). Membranes were blocked in 5% bovine serum albumin in tris‐buffered saline and tween buffer for 1 h, and the membranes were incubated with anti‐*p*‐Drp1^ser616^, Mfn1 and OPA1 overnight at 4°C. Actin (1:1000 dilution; Cell Signaling Technology) was used as a housekeeping protein. Bound antibodies were detected using horseradish peroxidase‐conjugated with anti‐mouse or rat IgG (1:200 dilution; Cell Signaling Technology). The membranes were exposed to an ECL western blotting substrate (Bio‐Rad Laboratories), and the densitometric analysis was carried out using a ChemiDoc Touch Imaging System (Bio‐Rad Laboratories).^15^

### 2.6 Inflammatory markers parameters

Blood was used to determine the inflammatory cytokines mRNA expression in white blood cells. The expression of TNF‐α and IL‐6 mRNA levels was analysed by SYBR Green real‐time Quantitative Reverse Transcription‐Polymerase Chain Reaction (qRT‐PCR) using mRNA extracts of the buffy coated blood. The thermal cycling condition was used to denaturation at 95°C for 20 s, alignment at 54°C for 20 s and elongation at 72°C for 20 s, for 40 cycles. The following primers were used in this study: (1) beta‐actin: forward, 5′‐CCAGATCATGTTTGAGACC‐3′ and reverse, 5′‐ATGTCACGCACGATTTCCC‐3′, (2) TNF‐α: forward, 5′‐GCTGCACTTTGGAGTGATCG‐3′ and reverse, 5′‐CTTACCTACAACATGGGCTACAG‐3′ and (3) IL‐6: forward, 5′‐GCTTGAATCTAAATTATCAGTC‐3′ and reverse, 5′‐GAAGATTCAAATTGCATCTTAT‐3′.^14^

### 2.7 Statistical analysis

All data were expressed as the mean ± SD or median (interquartile range) for continuous variables and percentage for categorical variables. A repeated one‐way anova followed by an LSD post hoc test was applied to test the effects of contrast media on renal function, circulating mitochondrial function and cell death. For subgroup analysis, a two‐way anova followed by an LSD post hoc test was used to test the temporal effects of contrast media in both non‐AKI and AKI groups. The differences between groups of categorical variables were compared by the chi‐square test or Fisher's exact test. Non‐parametric variables obtained at different times were compared using the median or Wilcoxon signed‐rank test. A *p*‐value less than 0.05 was considered statistically significant. All data were analysed with program STATA version 16.0 (StataCorp LLC).

## 3 RESULTS

### 3.1 Demographic and clinical data of patients

Demographic data of the patients are shown in Table 1. Of 20 CKD patients, 65% are men with the mean age of 73.0 ± 8.9 years. Risk factors for CI‐AKI including diabetes mellitus (DM), hyperuricaemia and heart failure were 65%, 30% and 35%, respectively. For CKD staging in this group of patients, CKD stages 3 and 4 were found in 85% and 15%, respectively. Estimated contrast volume used was 73.3 ± 8.9 mL.

**TABLE 1:** Demographic parameters of the CKD patients.

Characteristics	*n* = 20
Age (year)^a^	73.0 ± 8.9
Male^a^	13 (65.0)
Body weight (kg)^a^	60.2 ± 12.9
BMI (kg/m^2^)^a^	23.5 ± 3.9
Systolic BP^a^	128.5 ± 21.2
Diastolic BP^a^	72.8 ± 12.3
*Comorbidity* ^b^	
DM	13 (65.0)
Hypertension	17 (85.0)
Dyslipidaemia	12 (60.0)
Hyperuricaemia	6 (30.0)
Arrhythmia	5 (25.0)
Heart failure	7 (35.0)
Malignancy	1 (5.0)
Smoking	9 (45.0)
CKD	20 (100.0)
Stage 3	17 (85.0)
Stage 4	3 (15.0)
Stage 5	0 (0.0)
*Cause of CKD* ^b^	
DM	13 (65.0)
Unknown	7 (35.0)
*Medications* ^b^	
ACEI	5 (25.0)
ARB	5 (25.0)
𝛽‐blocker	12 (60.0)
CCB	5 (25.0)
α‐blocker	1 (5.0)
Diuretics	8 (40)
Nitrate	1 (5.0)
Warfarin	3 (15.0)
*CAG data* ^b^	
CAD lesion	1 (5.0)
Left main	16 (80.0)
LAD	15 (75.0)
LCX	13 (65.0)
RCA	9 (45.0)
*PCI procedure*	
POBA	1 (11.1)
DES	8 (88.9)
Contrast volume (mL)	73.3 ± 38.6
*Laboratory findings*	
Haemoglobin (g/dL)	11.8 ± 2.0
Haematocrit (%)	35.6 ± 5.5
WBC (mm^3^)	8631 ± 2605
Platelet (mm^3^)	225,100 ± 77,535
Blood sugar (mg/dL)	156.7 ± 48.5
BUN (mg/dL)	26.6 ± 11.9
Creatinine (mg/dL)	1.5 ± 0.5
GFR (mL/min/1.73 m^2^)	44.8 ± 11.8
Sodium (mmol/L)	139.0 ± 2.8
Potassium (mmol/L)	4.2 ± 0.7
Chloride (mmol/L)	103.7 ± 4.3
Bicarbonate (mmol/L)	22.4 ± 3.9
Calcium (mg/dL)	9.1 ± 0.6
Phosphorus (mg/dL)	3.4 ± 0.9
Magnesium (mEq/L)	1.7 ± 0.2
Uric acid (mg/dL)	7.6 ± 2.6
Albumin (g/dL)	3.8 ± 0.6
ALP (U/L)	107.2 ± 42.6
AST (U/L)	76.8 ± 133.4
ALT (U/L)	38.1 ± 57.4
TB (mg/dL)	0.5 ± 0.2
DB (mg/dL)	0.2 ± 0.2
INR	1.3 ± 0.4
PTT ratio	1.0 ± 0.2

Abbreviations: ACEI, angiotensin‐converting enzyme inhibitors; ALP, alkaline phosphatase; ALT, alanine aminotransferase; ARB, angiotensin receptor blockers; AST, aspartate aminotransferase; BMI, body mass index; BP, blood pressure; BUN, blood urea creatinine; CAD, coronary artery disease; CAG, coronary artery angiography; CCB, calcium channel blockers; CKD, chronic kidney disease; DB, direct bilirubin; DES, drug eluting stent; DM, diabetes mellitus; GFR, glomerular filtration rate; INR, international normalized ratio; LAD, left anterior descending coronary artery; LCX, left circumflex artery; PCI, percutaneous coronary intervention; POBA, plain old balloon angioplasty; PTT, partial thromboplastin time; RCA, right coronary artery; TB, total bilirubin; WBC, white blood cell.

^a^ Mean ± SD.

^b^ *n* (%).

### 3.2 Circulating mitochondrial dysfunction occurred prior to the rising of plasma and urine NGAL in CKD patients after receiving a contrast media

**FIGURE 2:**
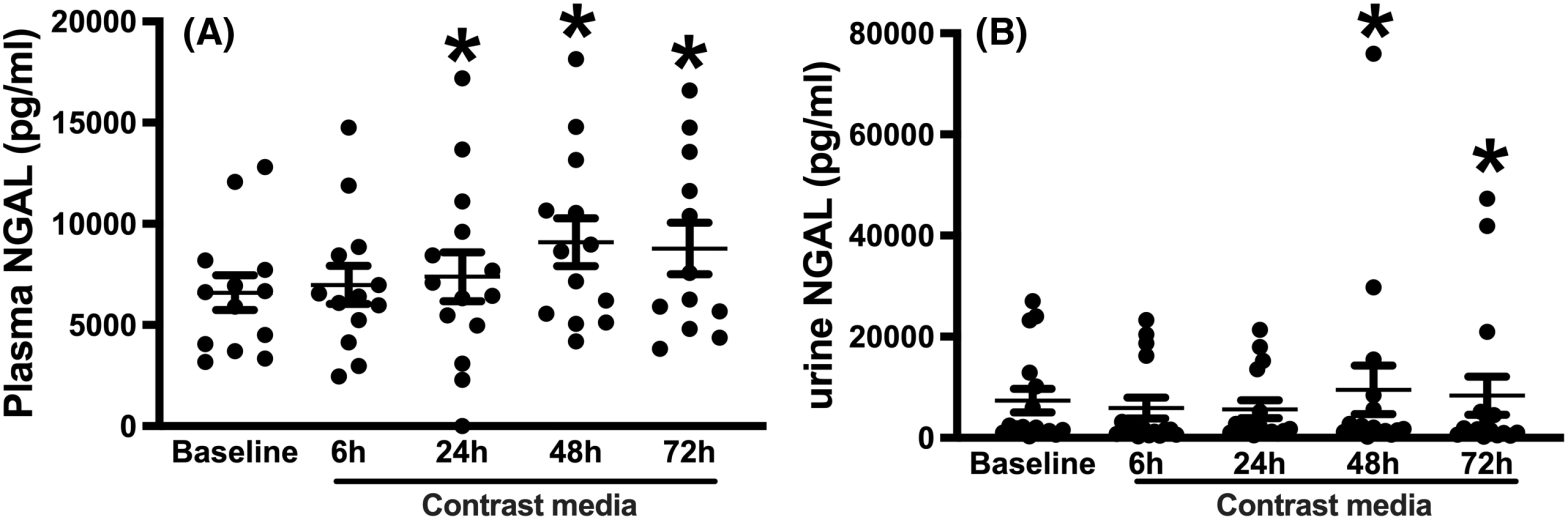
Levels of plasma and urine NGAL in CKD patients receiving contrast media during PCI. (A) Plasma NGAL levels in CKD patients receiving contrast media during PCI, (B) Urine NGAL levels in CKD patients receiving contrast media during PCI. *p < 0.05 vs. baseline. CI‐AKI, contrast‐induced acute kidney injury; CKD, chronic kidney disease; NGAL, neutrophil gelatinase‐associated lipocalin; PCI, percutaneous coronary intervention.

Our data demonstrated that plasma NGAL levels were upregulated at 24 h after receiving a contrast media (*p* < 0.05 vs. baseline), and the levels were persistent up to 72 h (*p* < 0.05 vs. baseline, Figure 2A). Since an increased urine NGAL levels were found later at 48 and 72 h after receiving a contrast media (*p* < 0.05 vs. baseline, Figure 2B), this suggested that the sensitivity of plasma NGAL was higher than urine NGAL in detecting AKI in CKD patient who receiving a contrast media.

**FIGURE 3:**
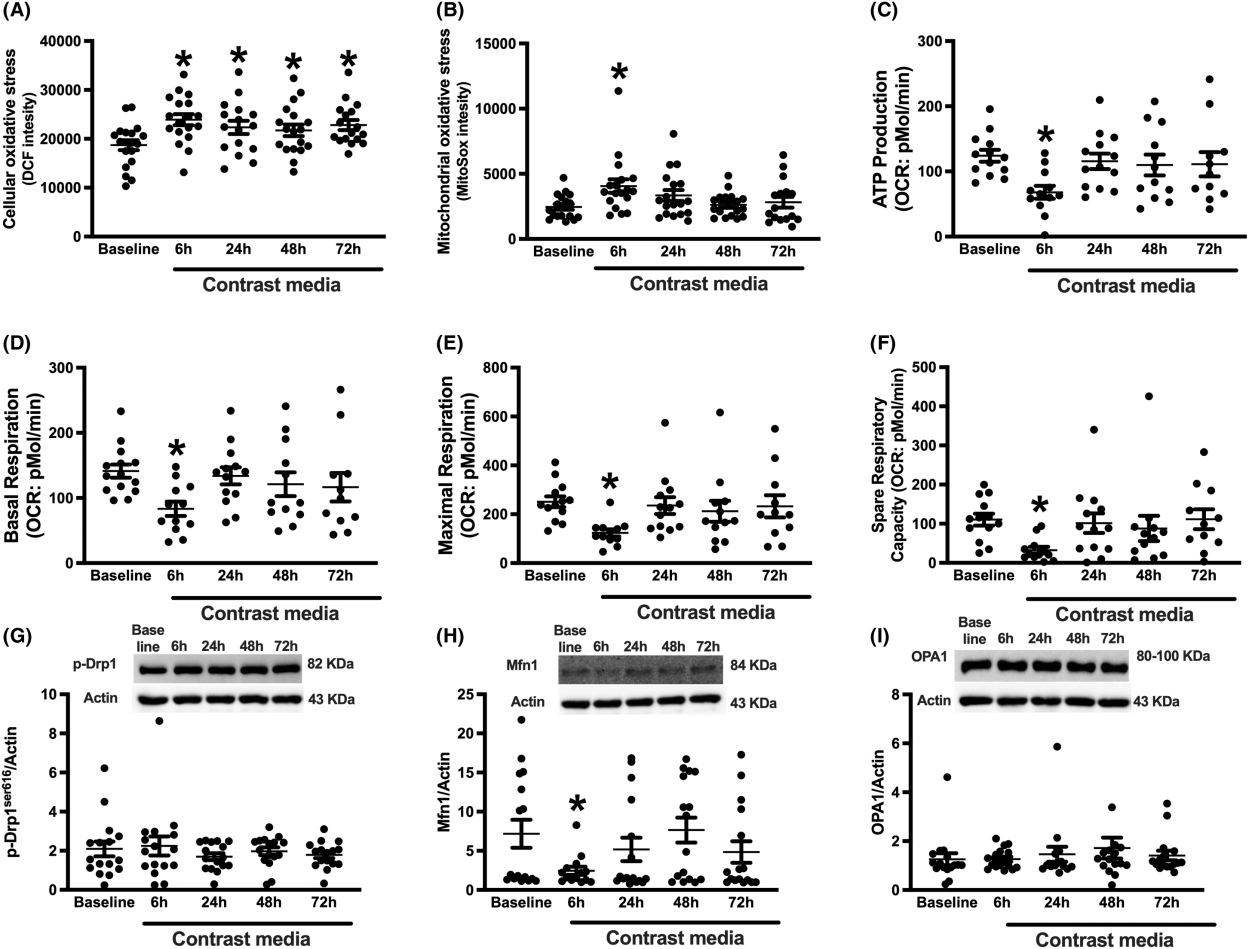
Levels of oxidative stress, mitochondrial respiration and mitochondrial dynamics in CKD patients receiving contrast media during PCI. (A) Cellular oxidative stress levels, (B) Mitochondrial oxidative stress levels, (C) ATP production, (D) Basal respiration, (E) Maximal respiration, (F) Spare respiratory capacity, (G) Mitochondrial fission marker (*p*‐Drp1^ser616^/Actin) with immunoblot analysis, (H) Mitochondrial fusion marker (Mfn1/Actin) with immunoblot analysis, (I) Mitochondrial fusion marker (OPA1/Actin) with immunoblot analysis. *p < 0.05 vs. baseline. ATP, adenosine triphosphate; CI‐AKI, contrast‐induced acute kidney injury; CKD, chronic kidney disease; DCF, dichlorofluorescein; Drp1, dynamin related protein 1; Mfn1, mitofusin 1; OCR, oxygen consumption rate; OPA1, optic atrophy protein 1; PCI, percutaneous coronary intervention.

Circulating mitochondrial function and dynamics were investigated in the isolated PBMCs from patients, and the results showed that cellular oxidative stress as measured by DCF fluorescent intensity was significantly increased at 6, 24, 48 and 72 h after receiving the contrast media (*p* < 0.05 vs. baseline, Figure 3A). These results suggested that cellular oxidative stress occurred at 6 h and continued to be high up to 72 h after receiving a contrast media.

At the mitochondrial level, the results showed that mitochondrial oxidative stress levels were increased at 6 h (*p* < 0.05 vs. baseline, Figure 3B); then, this levels gradually decreased at 24, 48 and 72 h after receiving a contrast media (*p* = 0.1, 0.8, 0.5 vs. baseline, Figure 3B). This trend was found in other mitochondrial parameters including mitochondrial respiration and mitochondrial fusion. For mitochondrial respiration parameters, our data demonstrated that ATP production, basal respiration, maximal respiration and spare respiratory capacity were suppressed at 6 h after receiving a contrast media (*p* < 0.05 vs. baseline, Figure 3C–F). Interestingly, these parameters returned to their physiological status after 24, 48 and 72 h after receiving a contrast media (Figure 3C–F).

The protein analysis of mitochondrial dynamics was investigated in this study, and our data showed that the contrast media did not affect *p*‐Drp1^ser616^ and OPA1 (Figure 3G,I and Figure S1A–C); however, it suppressed Mfn1 protein expression only at 6 h after receiving a contrast media (*p* < 0.05 vs. baseline, Figure 3H and Figure S1A,D). Collectively, these results indicated that a contrast media induced a short‐term circulating mitochondrial dysfunction, which in turn leads to a prolonged cellular oxidative stress.

### 3.3 Neither circulating inflammation nor cell death was observed in CKD patients after receiving the contrast media

**FIGURE 4:**
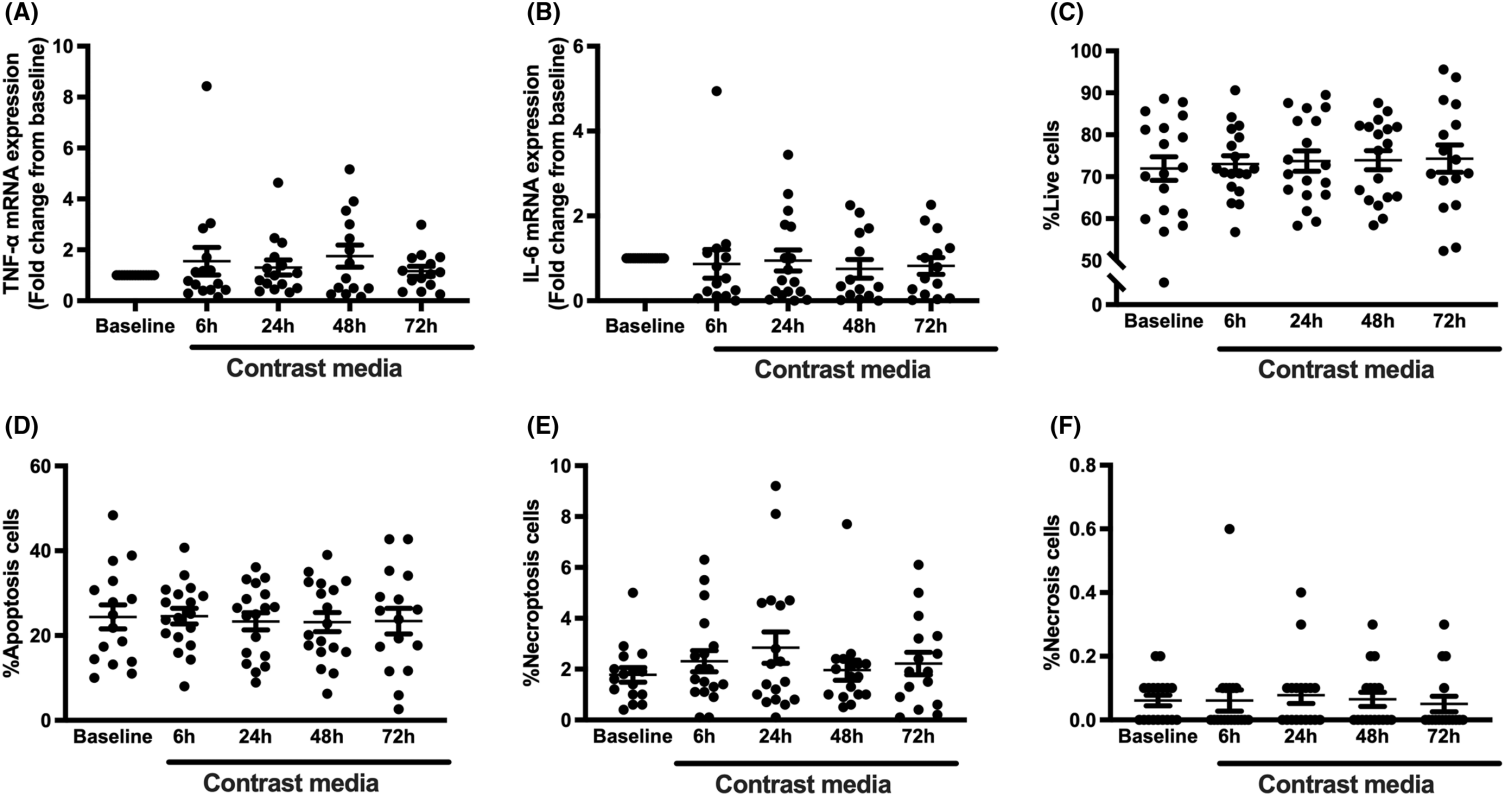
Levels of inflammatory cytokines and cell death in CKD patients receiving contrast media during PCI. (A) TNF‐α mRNA expression, (B) IL‐6 mRNA expression, (C) %Live cells, (D) %Apoptosis cells, (E) %Necroptosis cells, (F) %Necrosis cells. *p < 0.05 vs. baseline. CKD, chronic kidney disease; IL‐6, interleukin‐6; mRNA, messenger RNA; PCI, percutaneous coronary intervention; TNF‐α, tumour necrosis factor.

Inflammatory markers including TNF‐α and IL‐6 mRNA expression were investigated in the whole blood sample, and the results showed that the expression of both genes was not different between their baseline and among visits (Figure 4A,B). We further determined the types of cell death in PBMCs of these patients using annexin V/PI assay. Our data showed that the contrast media did not affect %live cells, %apoptosis cells, %necroptosis cells and %necrosis cells (Figure 4C–F). These results suggesting that a contrast media induce neither circulating inflammation nor cell death.

### 3.4 The incidence of CI‐AKI according to the serum Cr levels

To investigate whether CKD patients who developed CI‐AKI had worsen oxidative stress plasma and urine NGAL, mitochondrial functions, inflammation and cell death, we performed a subgroup analysis according to an increasing of serum Cr. Eight patients (40%) developed CI‐AKI during the study period. RRT was initiated in one patient (5%), and AKI‐related death was occurred in 1 patient (5%). For patients who did not develop AKI, serum Cr levels were not different from their baseline, suggesting that the renal function of these patients remained stable after contrast media administration. This information is shown in Table 2.

**TABLE 2:** Effects of contrast media on renal function.

Renal function	Baseline	6 h	24 h	48 h	72 h
Creatinine (mg/dL)	1.5 ± 0.6	1.6 ± 0.6	1.6 ± 0.6	1.7 ± 0.9	1.7 ± 0.9
BUN (mg/dL)	26.5 ± 11.9	27.9 ± 12.7	28.2 ± 13	28.6 ± 14.8	29.6 ± 13.4
eGFR (mL/min/1.73 m^2^)	44.8 ± 11.8	41.9 ± 12.5	44.1 ± 17.4	42.9 ± 18.2	42.1 ± 17.9

*Note*: Mean ± SD.

Abbreviations: BUN, blood urea nitrogen; eGFR, estimated glomerular filtration rate.

### 3.5 Plasma NGAL levels were increased in CKD patients who developed CI‐AKI after receiving a contrast media

**FIGURE 5:**
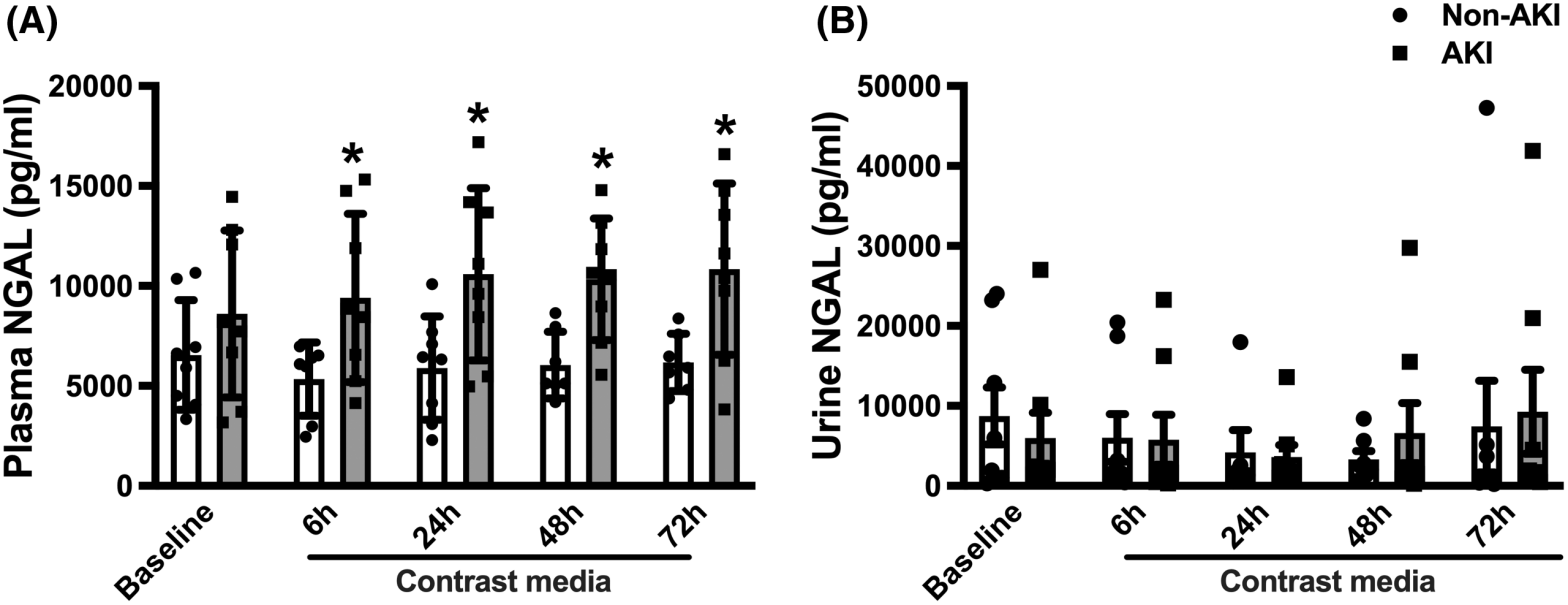
Levels of plasma and urine NGAL in CKD patients receiving contrast media during PCI who developed CI‐AKI vs. non‐AKI patients. (A) Subgroup analysis of plasma NGAL levels in CKD patients receiving contrast media during PCI who developed CI‐AKI vs. non‐AKI patients, (B) Subgroup analysis of urine NGAL levels in CKD patients receiving contrast media during PCI who developed CI‐AKI vs. non‐AKI patients. *p < 0.05 vs. baseline. CI‐AKI, contrast‐induced acute kidney injury; CKD, chronic kidney disease; NGAL, neutrophil gelatinase‐associated lipocalin; PCI, percutaneous coronary intervention.

In a subgroup analysis, plasma NGAL levels were significantly upregulated in CKD patients who developed CI‐AKI at 6, 24, 48 and 72 h after receiving a contrast media, and there was no different among visits (*p* < 0.05 vs. baseline, Figure 5A). Although urine NGAL levels were increased at 48 and 72 h, it was not statistically different among groups and visits (Figure 5B). Although plasma NGAL was not as good as mitochondrial parameters in early detecting the CI‐AKI in patients receiving contrast media, this subgroup analysis indicated that increased plasma NGAL could be used as a marker of CI‐AKI in CKD patients.

### 3.6 Cellular oxidative stress and mitochondrial alteration in CKD patients who developed CI‐AKI after receiving a contrast media

**FIGURE 6:**
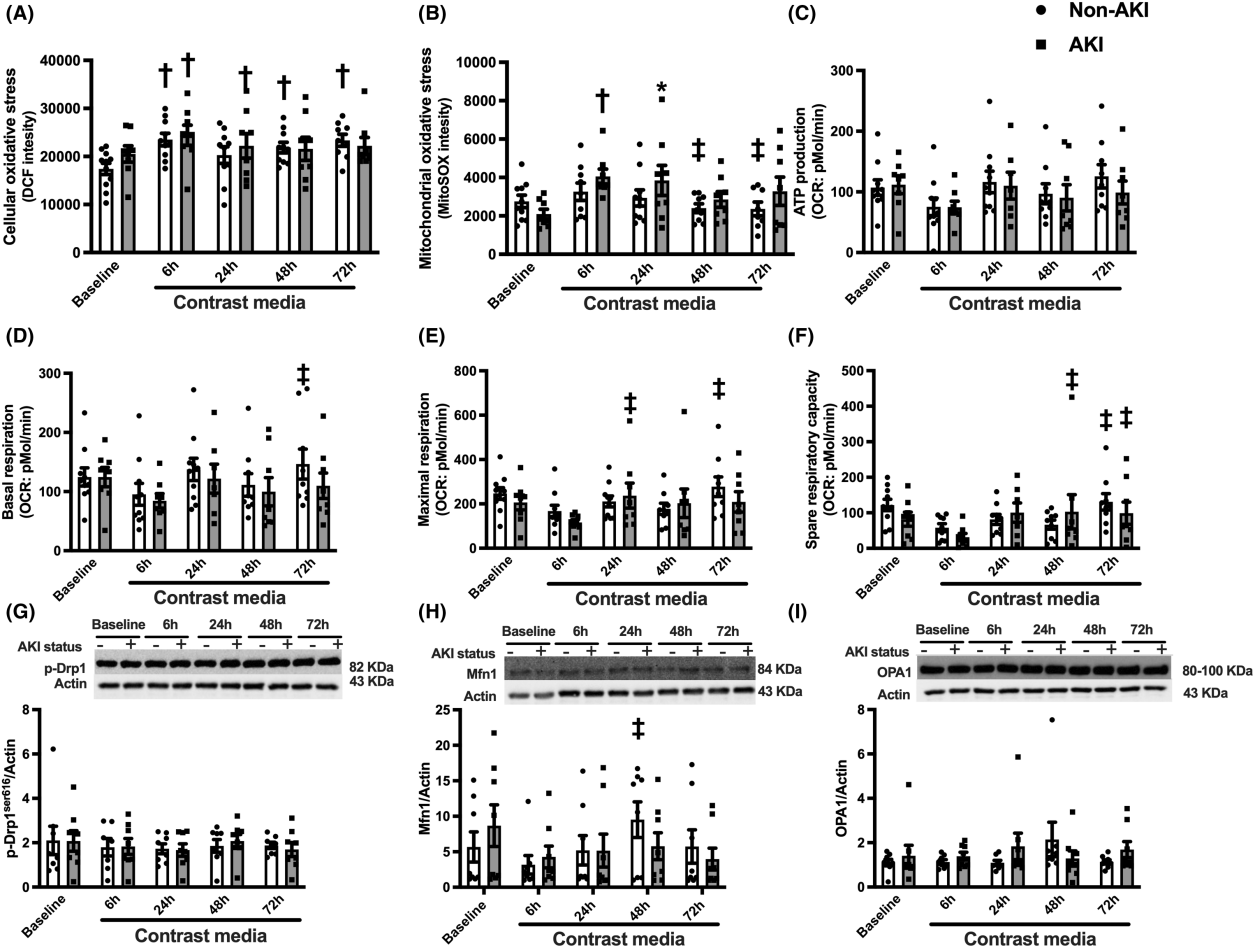
Subgroup analysis of the levels of oxidative stress, mitochondrial respiration and mitochondrial dynamics in CKD patients receiving contrast media during PCI who developed CI‐AKI vs. non‐AKI patients. (A) Cellular oxidative stress levels, (B) mitochondrial oxidative stress levels, (C) ATP production, (D) basal respiration, (E) maximal respiration, (F) spare respiratory capacity, (G) mitochondrial fission marker (*p*‐Drp1^ser616^/Actin) with immunoblot analysis, (H) mitochondrial fusion marker (Mfn1/Actin) with immunoblot analysis, (I) mitochondrial fusion marker (OPA1/Actin) with immunoblot analysis. *p < 0.05 vs. non‐AKI, ^†^ *p* < 0.05 vs. their respective baseline, ^‡^ vs. their 6 h value. ATP, adenosine triphosphate; CI‐AKI, contrast‐induced acute kidney injury; CKD, chronic kidney disease; DCF, dichlorofluorescein; Drp1, dynamin related protein 1; Mfn1, mitofusin 1; OCR, oxygen consumption rate; OPA1, optic atrophy protein 1; PCI, percutaneous coronary intervention.

For cellular oxidative stress, CKD patients who developed CI‐AKI had similar oxidative stress levels with those who did not develop CI‐AKI (Figure 6A). The rising of cellular oxidative stress in both groups were observed at 6 h after receiving a contrast media (*p* < 0.05 vs. baseline, Figure 6A). In the non‐CI‐AKI group, the cellular oxidative stress levels remain high until 72 h after receiving a contrast media (*p* < 0.05 vs. baseline, Figure 6A). In CI‐AKI group, the cellular oxidative stress levels increased at 6 and 24 h after receiving a contrast media (*p* < 0.05 vs. baseline, Figure 6A); then, the oxidative stress levels were reduced (*p* = 0.7, 0.5, Figure 6A).

At the mitochondrial level, mitochondrial oxidative stress levels were increased in CKD patients at 6 h after receiving a contrast media, especially in the CKD patients who developed CI‐AKI (*p* < 0.05 vs. baseline, Figure 6B). Although a statistical test showed no difference between baseline and 6 h in non‐CI‐AKI patients, mitochondrial oxidative stress levels were still at high level (*p* = 0.07 vs. baseline, Figure 6B). At 24 h, mitochondrial oxidative stress levels in CI‐AKI group remained to be higher than those of non‐CI‐AKI groups (*p* < 0.05; Figure 6B). At 48 and 72 h, in non‐CI‐AKI group, mitochondrial oxidative stress was significantly decreased, when compared to 6 h (*p* < 0.05; Figure 6B). These results suggested that the mitochondrial oxidative stress remained to be high longer in CI‐AKI group than that in non‐CI‐AKI group.

Regarding the mitochondrial respiration parameters, our results showed that, after receiving the contrast media, CKD patients with and without CI‐AKI exhibited similar levels of mitochondrial respiration (Figure 6C–F). However, we found that at 6 h mitochondrial respiration was suppressed in all groups, but it did not show a statistically significant. In non‐CI‐AKI group, at 72 h, basal respiration, maximal respiration and spare respiratory capacity were improved, when compared to 6 h (*p* < 0.05; Figure 6D–F). Additionally, in CI‐AKI group, maximal respiration was increased at 24 h, and spare respiratory capacity was improved at 48 and 72 h after receiving a contrast media, when compared with that at 6 h (*p* < 0.05; Figure 6D–F).

For mitochondrial dynamics, the subgroup analysis showed that *p*‐Drp^Ser616^ and OPA1 were not changed during a contrast media exposure (Figure 6G,I and Figure S1E–G). Nevertheless, the protein expression of Mfn1 was increased in non‐CI‐AKI group at 48 h after receiving a contrast media, when compared with 6 h (*p* < 0.05; Figure 6H and Figure S1E,H). These results suggested that mitochondrial alteration, by increased mitochondrial efficiency and promoted mitochondrial fusion, is one of the rapid response mechanisms to the contrast media exposure, which could be recovered to the physiological status within 48–72 h.

### 3.7 Circulating inflammation and necroptosis occurred in CKD patients who developed CI‐AKI after receiving a contrast media

**FIGURE 7:**
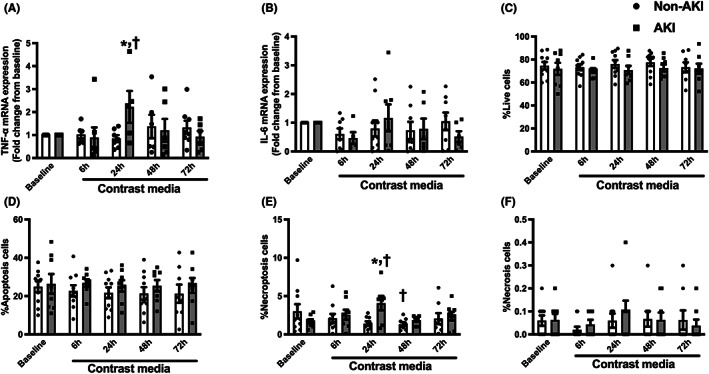
Subgroup analysis of the levels of inflammatory cytokines and cell death in CKD patients receiving contrast media during PCI who developed CI‐AKI vs. non‐AKI patients. (A) TNF‐α mRNA expression, (B) IL‐6 mRNA expression, (C) %Live cells, (D) %Apoptosis cells, (E) %Necroptosis cells, (F) %Necrosis cells. *p < 0.05 vs. non‐AKI, ^†^ *p* < 0.05 vs. their respective baseline. CI‐AKI, contrast‐induced acute kidney injury; CKD, chronic kidney disease; IL‐6, interleukin‐6; mRNA, messenger RNA; PCI, percutaneous coronary intervention; TNF‐α, tumour necrosis factor‐α.

In the subgroup analysis, TNF‐α mRNA expression was markedly increased in CI‐AKI group at 24 h after receiving a contrast media (*p* < 0.05 vs. baseline and non‐CI‐AKI group, Figure 7A). In contrast, IL‐6 mRNA expression, another inflammatory marker, was not different between groups and visits (Figure 7B). For cell death analysis, %live cells, %apoptosis cells and %necrosis cells were not different between groups and visits (Figure 7C,D,F). Interestingly, %necroptosis cells were increased at 24 h after receiving a contrast media (*p* < 0.05 vs. baseline and non‐CI‐AKI group, Figure 7E). These data indicate that an exposure of contrast media promoted circulating inflammation and necroptosis in CKD patients who had CI‐AKI after receiving a contrast media.

## 4 DISCUSSION

The results of our study in CKD patients demonstrated that after receiving a contrast media: (1) Plasma and urine NGAL levels were increased, but plasma NGAL was more sensitive to contrast media than urine NGAL, (2) Circulating oxidative stress, mitochondrial dysfunction and a decrease in mitochondrial fusion were evidenced following contrast media exposure and that the alteration of these mitochondrial function and dynamics occurred earlier than plasma NGAL, (3) The incidence of CI‐AKI was 40% according to the serum Cr levels, (4) In subgroup analysis, CKD patients who developed CI‐AKI had higher levels of plasma NGAL, circulating mitochondrial oxidative stress, inflammation and necroptosis than CKD patients without CI‐AKI. Furthermore, administration of a contrast media did not affect mitochondrial fission in the PBMCs from CKD patients.

The evidence of CI‐AKI has been established in patients receiving various types of contrast media during PCI procedure.^9^, ^17^, ^18^ In this study, we focused on the specific group of CKD patients, specifically at stages 3 and 4, who received only iodixanol as a contrast media. Previous studies in rats and in renal tubular epithelial cells reported that iodixanol caused mitochondrial dysfunction.^19^, ^20^ However, the roles of mitochondria in CI‐AKI in CKD patients are still unknown. Therefore, the molecular mechanisms involving the pathogenesis of CI‐AKI in CKD patients were investigated. First, we investigated the potential role of oxidative stress in modulating CI‐AKI in CKD patients. Since mitochondria are the major source of oxidative stress production,^21^ we performed an experiment to determined circulating mitochondrial function in PBMCs, and our results revealed that mitochondrial dysfunction occurred very early at 6 h after receiving a contrast media. During 6 h after receiving a contrast media, mitochondria experienced a transient low oxygen environment and, therefore, all mitochondrial respiration parameters were suppressed, while mitochondrial oxidative stress levels were increased. This is supported by a previous study demonstrating that a depletion of oxygen in medulla was observed in rabbits and rodents with CI‐AKI.^22^, ^23^ Since all mitochondria have a capability to adapt themselves to a stress environment via the mitochondrial dynamic control,^24^ these mitochondrial parameters returned to the physiological status at the 24 h (in the whole population) and at 48 h (in the AKI group) after contrast media exposure together with mitochondrial fusion that was suppressed at the 6 h and returned to normal status at the 24 h after receiving contrast media.

Although mitochondrial oxidative stress levels were increased only at 6 h after receiving a contrast media, it was high enough to be transported to the cytosol. Unlike mitochondria, cellular oxidative stress levels remained at the high levels from 6 to 72 h after receiving a contrast media. Excessive oxidative stress level could be one of potential causes of NGAL activation in our study. NGAL has been evaluated as potential marker of AKI after contrast media exposure or cardiac surgery.^25^, ^26^, ^27^ After the presence of circulating mitochondrial dysfunction and cellular oxidative stress, a rising of plasma NGAL levels were observed later at 24 h, followed by increased urine NGAL levels at 48 h after receiving a contrast media. NGAL has been proposed as another promising AKI biomarker in recent years.^28^ The relationship between NGAL and oxidative stress has been reported in a cellular study in which the expression of NGAL was significantly upregulated following oxidative stress stimulation, and the expression of NGAL was disappeared following an addition of antioxidant.^29^ These data suggested that cellular oxidative stress is a possible inducer of plasma NGAL release in our CKD patients after receiving a contrast media. Importantly, an increase in cellular oxidative stress along with increased plasma NGAL lasted until 72 h after receiving a contrast media. However, we did not observe the changes in the circulating inflammation and cell death in a whole population. Therefore, a subgroup analysis was performed according to the serum Cr levels.

By KDIGO criteria for AKI,^4^ we found 40% of our subjects developed CI‐AKI based on an increase in their serum Cr from baselines. In this subgroup analysis, our results showed that in addition to an increased mitochondrial oxidative stress and plasma NGAL, TNF‐α mRNA expression was also increased and associated with an increased %necroptosis cell in CI‐AKI patients. This observation was found only at 24 h after receiving a contrast media. TNF‐α was activated in this situation, and it is a key mediator of necroptosis.^30^ When TNF‐α binds to the receptor, the intracellular kinase including receptor interacting protein Kinase 1 and 3 (RIPK1/RIPK3) and mixed lineage kinase domain like pseudokinase (MLKL) were recruited, followed by a conformational changes of the receptors, leading to cell membrane rupture and a release of intracellular contents.^31^ In our CI‐AKI patient subgroup, necroptosis could be detected using Annexin V^+^/PI^+^ cells using a flow cytometry. However, a future study is needed to confirm the expression of MLKL, which is a final protein responsible for necroptosis.

**FIGURE 8:**
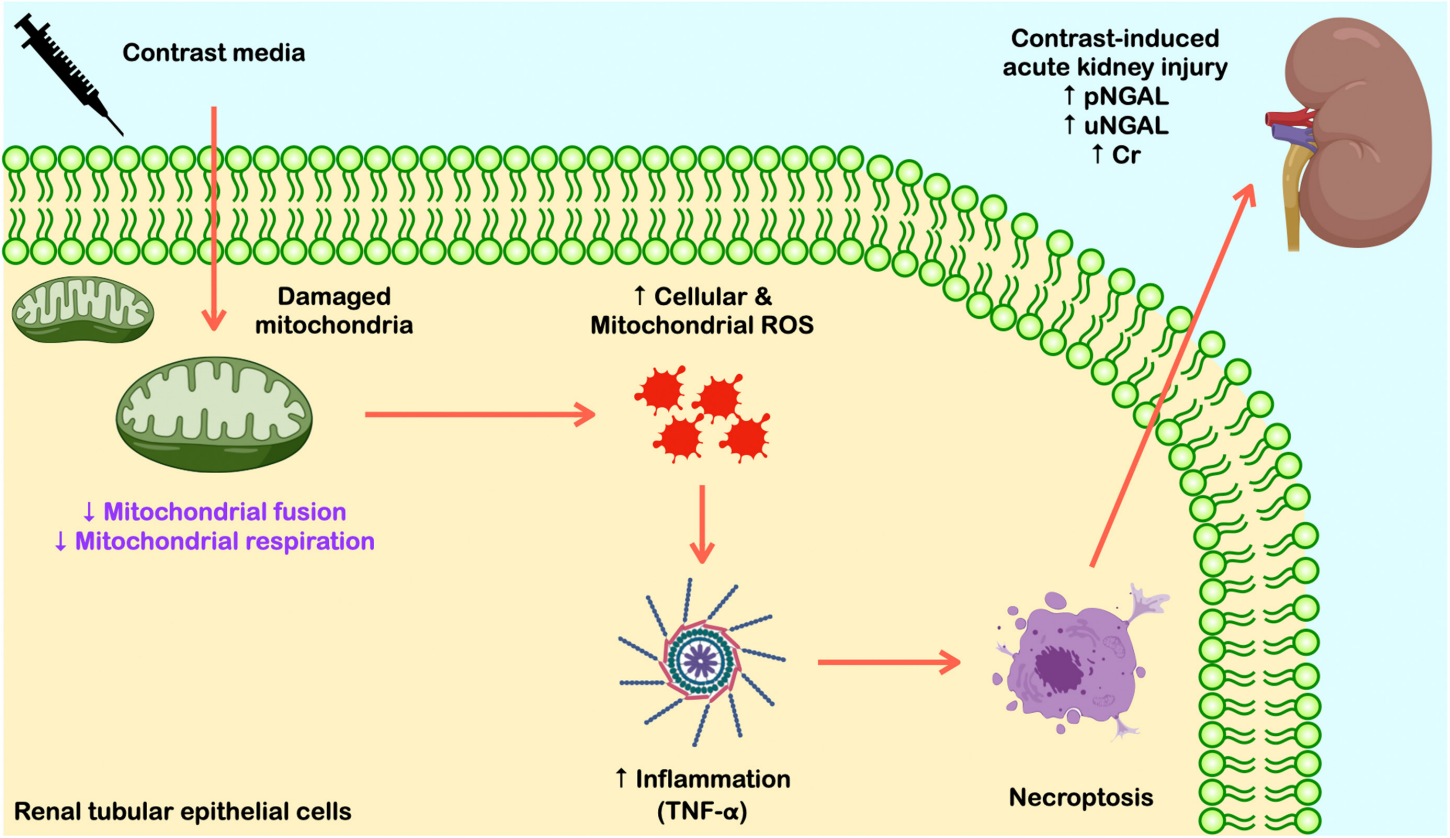
Mechanism of contrast‐induced AKI evidence from our study. CI‐AKI, contrast‐induced acute kidney injury; CKD, chronic kidney disease; NGAL, neutrophil gelatinase‐associated lipocalin; PCI, percutaneous coronary intervention; ROS, reactive oxygen species.

Collectively, our data indicated that mitochondrial dysfunction is the rapid response mechanism (at 6 h) following an administration of contrast media, which response to low oxygen environment and excessive oxidative stress production in CKD patients receiving contrast media during PCI. The oxidative stress is transported to the cytosol (6 h), initiated intracellular damage by activating NGAL (24 h), inflammation‐mediated necroptosis, and eventually leading to AKI in CKD patients. The summary of our study is demonstrated in Figure 8.

The strength of our study is that this is the first study to investigate the roles of mitochondrial function and mitochondrial dynamics in CKD patients with CI‐AKI. There are several limitations in this study. First, this study was conducted in a single centre, that led to a limited number of participants. Also, other early AKI biomarkers which are clinically approved for AKI and show a high sensitivity than NGAL, such as TIMP‐2∙IGFBP7, were not determined in this study. Since 8 of 20 patients had AKI following a contrast media exposure, renal function in 12 patients remained stable. This might be explained by a difference in antioxidant levels in each individual that might affect the renal susceptibility to a contrast media. However, we did not measure it in this study. Thus, antioxidant levels should be measured in a future study to prove this hypothesis.

## 5 CONCLUSIONS

We demonstrated that in CKD patients undergoing PCI, although plasma and urine NGAL are more sensitive to AKI after receiving contrast media compared with Cr, mitochondrial dysfunction and impaired mitochondrial fusion detected from PBMCs can be observed earlier than NGAL. Both cellular oxidative stress and NGAL also led to systemic inflammation and necroptosis later in the AKI subgroup. These findings may pave ways to devise strategies to prevent CI‐AKI by combating these sequential pathophysiologic paradigms.

## AUTHOR CONTRIBUTIONS

**Prit Kusirisin:** Conceptualization (equal); data curation (lead); formal analysis (equal); funding acquisition (equal); investigation (lead); methodology (lead); validation (equal); visualization (lead); writing – original draft (lead). **Nattayaporn Apaijai:** Data curation (equal); formal analysis (equal); investigation (equal); methodology (equal); validation (equal); visualization (equal); writing – review and editing (supporting). **Kajohnsak Noppakun:** Investigation (supporting); methodology (supporting); validation (supporting); visualization (supporting); writing – original draft (supporting). **Srun Kuanprasert:** Formal analysis (supporting); investigation (supporting); methodology (supporting); resources (supporting); validation (supporting); visualization (supporting); writing – original draft (supporting). **Siriporn Chattipakorn:** Funding acquisition (equal); investigation (equal); methodology (equal); validation (supporting); visualization (supporting); writing – review and editing (supporting). **Nipon Chattipakorn:** Conceptualization (equal); funding acquisition (lead); investigation (supporting); methodology (supporting); project administration (supporting); supervision (lead); writing – review and editing (lead).

## FUNDING INFORMATION

This work was supported by the NSTDA Research Chair grant from the National Science and Technology Development Agency Thailand (to NC); the Distinguished Research Professor grant from the National Research Council of Thailand (N42A660301 to SCC); the National Research Council of Thailand (N42A660432 to NA, N35A650175 to PK); the Chiang Mai University Center of Excellence Award (to NC) and the National Kidney Foundation of Thailand (to PK).

## CONFLICT OF INTEREST STATEMENT

The authors declare that they have no conflict of interests.

## Supporting information


Figure S1.

## Data Availability

The data sets generated and analysed during the current study are not publicly available due to the data confidentiality requirements of the ethics committee but are available from the corresponding author on reasonable request and approval from the ethics committee.
