# Supplementary material for: Circulating mitochondrial dysfunction as an early biomarker for contrast media‐induced acute kidney injury in chronic kidney disease patients
Source: J Cell Mol Med. 2023 Jun 12;27(14):2059–70. doi: 10.1111/jcmm.17806 (PMC10339076; doi:10.1111/jcmm.17806)
Supplement: Supplementary file 1 — Figure S1. [file JCMM-27-2059-s001.docx]

**Supplementary material**

**Supplementary Figure 1. Uncropped figures of the immunoblot analysis in mitochondrial dynamics.**

**(A-D) Immunoblot analysis of mitochondrial dynamics in chronic kidney disease (CKD) patients receiving contrast media during percutaneous coronary intervention (PCI);** (A) Actin, (B) Mitochondrial fission marker (p-Drp1^ser616^), (C) Mitochondrial fusion marker (OPA1), (D) Mitochondrial fusion marker (Mfn1)

**(E-H) Immunoblot analysis of mitochondrial dynamics in CKD patients receiving contrast media during PCI who developed contrast-induced acute kidney injury (CI-AKI) vs. non-AKI patients;** (E) Actin, (F) p-Drp1^ser616^, (G) OPA1, (H) Mfn1
